# A Simple and Cost-Effective FeCl_3_-Catalyzed Functionalization of Cellulose Nanofibrils: Toward Adhesive Nanocomposite Materials for Medical Implants

**DOI:** 10.1021/acsami.4c04351

**Published:** 2024-05-31

**Authors:** Evgenii Tikhomirov, Antonio Franconetti, Mathias Johansson, Corine Sandström, Elin Carlsson, Brittmarie Andersson, Nils P Hailer, Natalia Ferraz, Carlos Palo-Nieto

**Affiliations:** † Nanotechnology and Functional Materials, Department of Materials Science and Engineering, Uppsala University, Uppsala 751 03, Sweden; ‡ Departamento de Química Orgánica, Facultad de Química, Universidad de Sevilla, Sevilla 41012, Spain; § Department of Molecular Sciences, Swedish University of Agricultural Sciences, Uppsala 756 51, Sweden; ∥ Ortholab, Department of Surgical Sciences—Orthopaedics, Uppsala University, Uppsala 751 85, Sweden

**Keywords:** nanocellulose surface-chemistry, Lewis acid catalysis, nanocellulose-based composites, coating hydrogels, medical implants

## Abstract

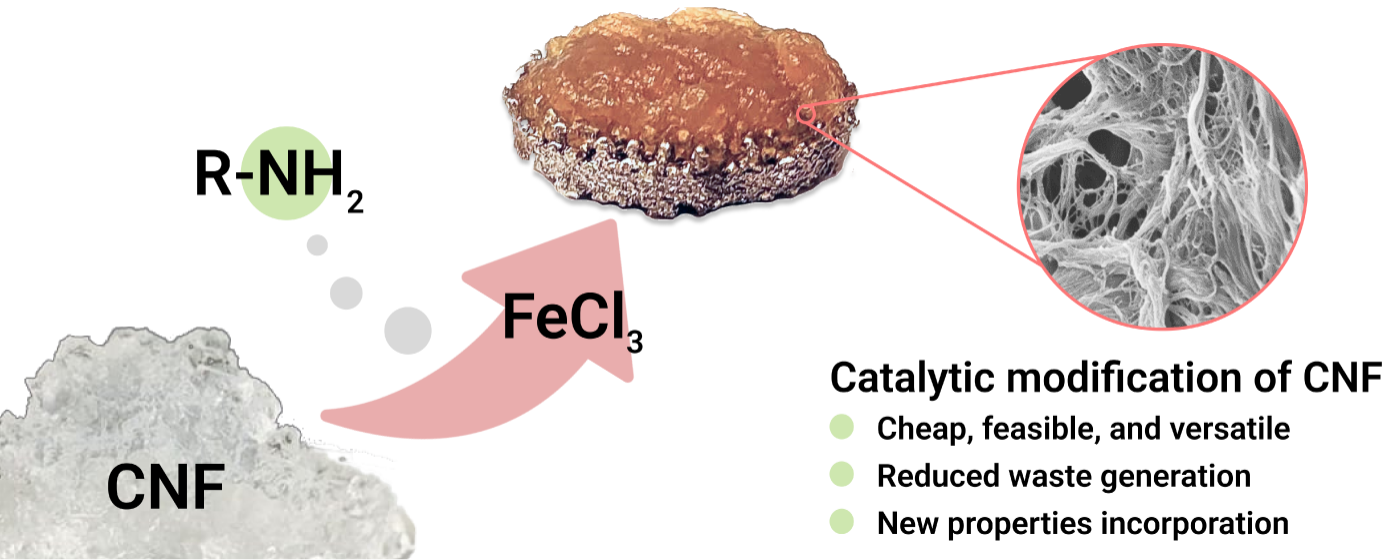

In the present work, we explored Lewis acid catalysis, via FeCl_3_, for the heterogeneous surface functionalization of cellulose nanofibrils (CNFs). This approach, characterized by its simplicity and efficiency, facilitates the amidation of nonactivated carboxylic acids in carboxymethylated cellulose nanofibrils (c-CNF). Following the optimization of reaction conditions, we successfully introduced amine-containing polymers, such as polyethylenimine and Jeffamine, onto nanofibers. This introduction significantly enhanced the physicochemical properties of the CNF-based materials, resulting in improved characteristics such as adhesiveness and thermal stability. Reaction mechanistic investigations suggested that endocyclic oxygen of cellulose finely stabilizes the transition state required for further functionalization. Notably, a nanocomposite, containing CNF and a branched low molecular weight polyethylenimine (CNF-PEI 800), was synthesized using the catalytic reaction. The composite CNF-PEI 800 was thoroughly characterized having in mind its potential application as coating biomaterial for medical implants. The resulting CNF-PEI 800 hydrogel exhibits adhesive properties, which complement the established antibacterial qualities of polyethylenimine. Furthermore, CNF-PEI 800 demonstrates its ability to support the proliferation and differentiation of primary human osteoblasts over a period of 7 days.

## 1 Introduction

Wood-derived cellulose nanofibrils (CNFs) have emerged as a sustainable material with a wide range of applications (such as biomedicine,^1^ electronics devices,^2^ food packaging^3^ and adsorbents^4^). Several processes have been developed to produce CNFs and they usually are combined with chemical and/or enzymatic pretreatments of the wood pulp to facilitate the fibrillation. As an example of chemical pretreatment, carboxymethyl groups can be introduced to the surface to produce carboxymethylated CNF (c-CNF).^5^ Due to the presence of the −COOH groups, c-CNF can be further modified in order to graft active molecules onto the nanofibers, e.g., via amide (−CONH−) bond formation.^6^ The surface modification of CNFs is a well-known approach to increase the applicability for industrial and biomedical applications.^7^

The number of synthetic approaches to modify CNFs has increased considerably during the last years.^8 −10^ With this increase, there is a need for greener, cheaper, and more efficient chemical procedures because of environmental concerns about the waste management of hazardous chemicals. In this context, heterogeneous modifications have been employed for the chemical modification of CNFs.^11^ These heterogeneous approaches offer several advantages over the homogeneous synthesis methods. First, they do not require complete solubilization of cellulose, simplifying the process and reducing dissolution challenges. Second, when other reagents are soluble in the reaction medium, purification is easier and more cost-effective. Additionally, the inherent structure of cellulose is better preserved, which is crucial for maintaining the desired properties of CNFs, making heterogeneous methods preferable in some applications.

As a drawback, heterogeneous modifications typically use stoichiometric or overstoichiometric concentrations of toxic reactants as well as acids or bases to activate nanocellulose,^12^ resulting in large volumes of waste.

Several publications have described the modification of the carboxylic acids in oxidized nanocellulose by amidation with an amine group, requiring coupling reagents like carbodiimide compounds.^13−15^ In some cases, a thermal condensation reaction can be achieved,^16,17^ but in practice, this approach is rarely employed due to relatively limited scope and high-temperature requirements. Additionally, there is the possibility of using methods that activate carboxylic acids followed by aminolysis and using toxic cross-linkers, such as glutaraldehyde and epichlorohydrin.^18^ In general, these methods produce wasteful byproducts and have poor atom economy. Therefore, new chemical procedures are needed to modify CNFs without using stoichiometric, toxic, and expensive reagents. Some synthetic methods from low-molecular-weight molecules, including catalysis, have been adapted for polysaccharide modification. For example, Lewis acid catalysts have been used previously for the acetylation of polysaccharides.^19^ Within this context, iron stands out as one of the most abundant metals on Earth, making it both cost-effective and environmentally friendly. In particular, FeCl_3_ has garnered special attention for its qualities of being economical, nontoxic, and environmentally benign.^20^

In addition, the development of polymer nanocomposites using CNFs as a nanofiller has increased in interest due to their exceptional properties, such as biodegradability.^21^ It is necessary to covalently or noncovalently link the polymer and nanocellulose in order to accomplish the desired functionalities. Diverse chemical procedures are described to incorporate the polymer into the CNF with covalent interactions, i.e., silane coupling, etherification, esterification, or amidation.^22−24^ The type of interaction and degree of incorporation are the most important factors for the final properties of the nanocomposite. Therefore, in this work, we investigated the covalent incorporation of amine-containing polymers to CNFs using a cost-effective catalyzed procedure, as a way to improve their physicochemical properties, increasing their value for diverse applications.

One significant application of biomaterials involves the use of natural and synthetic polymers for antibacterial coatings of medical implants.^25^ Medical devices such as those used in total joint arthroplasty (TJA) are not sufficiently protected by the immune system since they are foreign bodies, and adherence of only a few bacteria can result in catastrophic infections. Infections of TJA components are defined as periprosthetic joint infection (PJI), and PJI is the primary cause of early TJA failure.^26,27^ To address this challenge, surface modifications of implants have emerged as a promising strategy to inhibit bacterial colonization and biofilm formation.^28^ Crucially, any coating intended for use in this context must strike a delicate balance, offering effective protection against bacteria while still enabling the process of osseointegration by osteoblast ongrowth. Nanocellulose, in various forms such as aerogel and hydrogel, has been investigated for the treatment of bone-related diseases and shows potential in this context.^29−31^

Additionally, polyethylenimine (PEI), a cationic polymer, exhibits promising antibacterial activity against both Gram-positive and Gram-negative bacteria^32−34^ but poses cytotoxicity concerns.^35,36^ Efforts to mitigate PEI’s cytotoxicity through chemical modifications have been explored, but achieving consistent outcomes remains a challenge.^37−39^ An ideal coating material for PJI prevention should possess the ability to integrate biomolecules with antibacterial activity and low cytotoxicity to human cells. Previously, PEI has been integrated into nanocellulose for a range of applications, including the production of biosorbents, packaging materials, and more.^40−43^

This study has several overarching goals. Initially, we aimed to investigate a cost-efficient chemistry, specifically metal catalysis, for the functionalization of CNFs while optimizing the associated conditions. Subsequently, the focus shifts to exploring the potential application of the resulting CNF-PEI 800 material as a coating material for the prevention of PJI. The hydrogel must demonstrate stand-alone potential, the ability to integrate additional biomolecules, and low cytotoxicity to human cells, all essential qualities for its intended use as an effective coating material in PJI prevention.

## 2 Materials and Methods

### 2.1 Chemical and Reagents

c-CNF (carboxyl group content 1800 μmol/g dry CNF) was purchased from RISE Bioeconomy (Stockholm, Sweden) and produced by the method described by Hua et al.^44^ 1,8-Diaminooctane, toluene (anhydrous, 99.8%), glacial acetic acid (AcOH), 2-methyltetrahydrofuran (2-MeTHF), cyclopentyl methyl ether (CPME), benzylamine, FeCl_2_, FeCl_3_, Fe_3_Br, Fe(III) *p*-toluenesulfonate hexahydrate ((CH_3_C_6_H_4_SO_3_)_3_Fe·6H_2_O), iron(III) oxide (Fe_2_O_3_), PEI (branched, Mw aprox. 800 g/mol), and α,x-diamino-terminated poly(oxypropylene)-*block*-poly(oxyethylene)-blockpoly(oxypropylene) (Jeffamine ED series) with an average molecular weight of 600 and 2003 g/mol were purchased from Sigma-Aldrich Sweden AB, Sweden, and used as received.

### 2.2 CNF Surface Modification Using Metal Catalysis

#### 2.2.1 General FeCl_3_-Catalyzed Amidation Procedure

An aqueous suspension of c-CNF (15 g of 2 wt % suspension, 0.3 g of dry *c*-CNF, 0.54 mmol of COOH) was solvent exchanged to acetone and then to toluene. c-CNF was then resuspended in dry toluene (20 mL), and FeCl_3_ (0.054 mmol, 10 mol %) and AcOH (15 μL, 0.5 equiv) were added and the reaction mixture was vigorously stirred at 50 °C for 10 min. Then, benzylamine or amine-containing polymer (1.62 mmol, 3 equiv) was added to the reaction mixture and vigorously stirred for 16 h at 110 °C. Afterward, the mixture was cooled to 25 °C and the solvent was replaced with acetone and then water. For purification, the modified material was washed once with a 0.1 M HCl solution and twice with 0.1 M NaOH/0.05 M NaHCO_3_ buffer (pH 11) in order to remove any unreacted amine potentially entrapped within the nanofibers, as well as impurities. Dialysis against deionized water was then performed until the conductivity in water was <0.005 mS/cm^2^.

### 2.3 CNF-Based Material Characterization

#### 2.3.1 Determination of Benzylamine and Copolymer Content on the CNF Surface

The benzylamine and copolymer content in the functionalized CNF materials were determined by elemental analysis of total nitrogen content. The technique used for the determination of CHN is based on the quantitative dynamic flash combustion method. CNF-copolymer freeze-dried samples were submitted to MEDAC LTD Analytical and chemical consulting services in Chobham (United Kingdom). The Thermo FlashEA^R^ 1112 instrument was used for nitrogen quantification. The weight percentage of nitrogen in CNF-copolymer was converted to mmol copolymer/g CNF.^45^

#### 2.3.2 Chemical Structure of CNF-PEI 800 Nanocomposite Material

The chemical structure of the CNF-PEI 800 material was characterized by solid-state nuclear magnetic resonance (NMR). The NMR spectra was obtained on a Bruker Avance III 600 MHz spectrometer using a double-resonance 4 mm (^1^H and ^19^F)/(^15^N–^31^P) CP-MAS probe and 4 mm ZrO_2_ rotors. The ^13^C cross-polarization (CP) magic angle spinning (MAS) NMR spectra were recorded at a spinning frequency of 12 kHz, a contact time of 1–2 ms, and a repetition delay of 3–5 s. The experiments were performed at 25 °C.

#### 2.3.3 Adhesiveness of CNF-PEI 800 Nanocomposite Material

The adhesiveness of the c-CNF and CNF-PEI 800, both 1.5 wt %, were evaluated using a Discovery HR3 rheometer (TA Instruments, New Castle, DE, USA) equipped with a 40 mm diameter aluminum parallel plate. A lower-styrene Peltier plate was used to control the temperature of the sample. Measurements were performed at 25 °C. Samples were loaded with a gap of 1 mm. A 300 s soak time with a controlled compression force of 0.1 ± 0.1 N was applied before the measurement. The gap was increased to 20 mm at a speed of 0.5 mm/s. The apparent adhesiveness was evaluated as the maximum force (N) recorded during the measurement. The apparent work of adhesion was evaluated as the area under the curve (N·μm) when plotting the force (N) vs gap (μm). Measurements were performed in triplicates. Additional measurements were performed to evaluate the adhesiveness on titanium surfaces relevant for medical application. The measurements were performed as outlined above but with a titanium-scaffold (12.5 mm diameter) attached to the lower and upper geometry of the instrument and the sample loaded between the scaffolds.

#### 2.3.4 Surface Morphology of the CNF-PEI 800 Nanocomposite Material

The surface morphology of the CNF-based materials was examined by scanning electron microscopy (SEM), while SEM with an energy dispersive spectroscopy (EDS) system was used to observe the distribution of the PEI polymer and the metal-catalysts on the CNF material surface. The samples were prepared by resuspending the materials in tetrahydrofuran, placing a drop of the suspension on a glass slide, and allowing it to dry at room temperature. Afterward, the samples were mounted on carbon stubs and coated with a conductive thin layer (3–4 nm) of gold and palladium using a Polaron SC7640 sputter coater (Quorum Technologies Ltd., Newhaven, UK). Samples were imaged using a Zeiss Merlin SEM with an SE2 detector and an energy-dispersive detector EDS (Carl Zeiss Microscopy, Oberkochen, Germany).

#### 2.3.5 Statistical Analysis

Significant differences (*p* < 0.05) between samples were determined by a two-sample *t* test after ensuring that the assumption of near-normal data distribution was fulfilled, using Minitab (Minitab 18.1, Minitab LCC, State College, PE, USA).

### 2.4 CNF-PEI 800 Nanocomposite Material Biological Characterization

#### 2.4.1 Cell Cultures with Primary Human Osteoblasts

The human osteoblasts (hOBs) were isolated from human femoral heads after assessment by the Swedish Ethical Review Authority (approval number: 2020–04462) following previously published protocols.^46^ The obtained bone parts were finely cut into 1–2 mm fragments, rinsed with phosphate-buffered saline (PBS, Gibco) and placed for expansion in 25 cm^2^ flasks supplemented with complete media (CM) containing α-MEM, 10% fetal bovine serum (FBS, Merck, KGaA, Darmstadt, Germany), 1% penicillin/streptomycin, and 0.5% amphotericin. After 4 weeks, the cells were transferred to 75 cm^2^ flasks and further expanded until passages 3 to 6 for the cell culture experiments.

CNF-PEI 800 and c-CNF hydrogels (1.5 wt %) were placed into individual wells of a 24-well plate, with each well containing 0.12 g of the hydrogel material. To ensure sterility, the hydrogels were subjected to the sterilization process, which involved two sequential washes of 20 min each with 70% ethanol (EtOH), followed by two additional washes with phosphate-buffered saline (PBS). Following sterilization, the hydrogels were preincubated overnight with complete media (CM). The next day, 35 000 cells were seeded into each well, along with the hydrogel scaffolds, using 100 μL droplets. The total volume in each well was adjusted to 1 mL by adding an additional 900 μL of the medium. Three technical replicates were performed per hydrogel at every time point. Cell proliferation and any signs of cytotoxicity of the hydrogels were assessed at 3 and 7 days. Images were captured using a Plan-Apochromat 10 × /0.45 objective on a Zeiss microscope.

#### 2.4.2 Cell Morphology

The morphology of the cells seeded in the wells on c-CNF and CNF-PEI 800 hydrogels was visualized by staining the cytoplasm with carboxyfluorescein diacetate (CFDA; Merck KGaA). The cells were fixed with 4% (v/v) paraformaldehyde at room temperature for 20 min, rinsed three times with PBS, and permeabilized using 0.1% Triton X-100 (Merck KGaA) for 15 min. The cytoplasm was stained with CFDA (500 nM) for 15 min, and then normal 10% goat serum (s-1000; Sigma-Aldrich, Sweden) blocking solution in PBS with 2% bovine serum albumin (BSA) and 0.3% Triton X-100 for 30 min. The side of the hydrogels that was seeded with cells was placed face down in a dish with a glass coverslip bottom (ibidi, Munich, Germany) for imaging, keeping it hydrated with PBS. Images were obtained using a Plan-Apochromat 10 × /0.45 (Zeiss) objective. Cells seeded on hydrogels were imaged with a pinhole setting of 2 Airy units (14-μm sections) for every Z section over the total height where cells were distributed in the scaffolds.

#### 2.4.3 Cell Viability

A lactate dehydrogenase enzymatic assay (LDH, TOX7, Merck, KGaA, Darmstadt, Germany) was used to quantify cell adhesion and proliferation after 7 days. The hydrogels with cells were rinsed with PBS (×1), transferred to new wells, and lysed with 400 μL of lysis buffer (CellLytic, Sigma-Aldrich, Sweden) for 15 min at 300 rpm on a shaker. Following manufacturer’s protocol, 50 μL of cell lysate was mixed with TOX7 reagents, and the absorbance was measured at 690–490 nm in a spectrophotometer (Multiscan Ascent, ThermoFisher Scientific Inc., Waltham, MA). The absorbance at 690 nm was subtracted from the absorbance at 492 nm, and the resulting values were expressed as LDH/cm^2^ in comparison with control values (c-CNF coated wells).

### 2.5 Computational Details

Theoretical calculations were carried out following a DFT scheme at the ωB97x-D/6-31G(d,p) level of theory (see Supporting Information for more information) using the Gaussian 16 software. Upon optimization, an analysis of frequencies was performed. Minima structures were characterized by the absence of imaginary frequencies, whereas transition states have exclusively one imaginary value (Nimag = 1). In all calculations, implicit solvation (toluene) was considered as a continuous isotropic medium by using the IEF-PCM model. This model creates solute cavities by overlapping spheres. Then, the electron density of the solute is employed to polarize the continuum. On the other hand, additional four explicit amine molecules were also added in these calculations.

## 3 Results and Discussion

### 3.1 Material Synthesis and Characterization

**Scheme 1:**
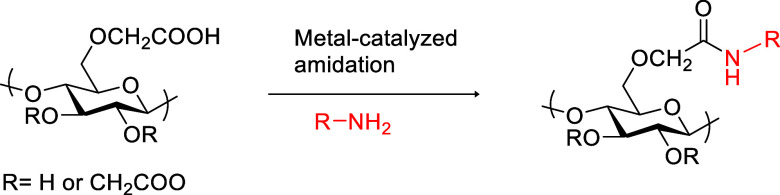
Metal-Catalyzed Functionalization of c-CNF

Metal chlorides have found application in the catalytic modification of polysaccharides.^47^ However, there is a lack of catalytic procedures for the grafting of amine-containing molecules onto polysaccharides in general and specifically onto CNFs. In this work, we have optimized a catalytic chemical procedure using a commercially available and cheap iron (FeCl_3_) salt (Scheme 1). Iron as catalyst has received attention because of its low cost, good reactivity, good stability under air, and nontoxicity.^48^ The reactions were carried out in toluene (PhMe) as solvent, which has been previously used for chemical modification of CNF.^49,50^ For the optimization of the catalytic direct amidation, benzylamine was used as the model molecule, a primary alkyl amine, which is frequently used as a nucleophile to validate new chemical conditions.^51^

Initial experiments began with the screening of the reaction using FeCl_3_ as the catalyst. Excitingly, as summarized in Table 1, the reaction employing 20 mol % of the catalyst in toluene at 110 °C (entry 2), resulted in the incorporation of benzylamine with a yield of 0.38 mmol benzylamine/g CNF. Reducing the FeCl_3_ concentration to 10 mol % did not compromise the reaction yield (entry 3). The introduction of AcOH glacial as an additive yielded a higher benzylamine incorporation (entry 4, 0.47 mmol of benzylamine/g of CNF) compared to the traditional EDC/NHS coupling method (0.34 mmol of benzylamine/g of CNF). To verify that the amidation reaction was indeed catalyzed by the metal-catalyst, the reaction was performed without the catalyst at 110 °C. The amount of benzylamine incorporated in this case was not detectable by elemental analysis (entry 1). It is noteworthy that a decrease of loading catalyst from 10 to 5 mol % (entry 5) was translated into a decrease in the benzylamine incorporation. Despite the observed decrease in incorporation at lower temperatures, it is significant that the reaction remains effective at 50 °C. This capability may offer a distinct advantage when considering the incorporation of temperature-sensitive molecules (entry 6). Next, a control experiment was carried out using 0.5 equiv of AcOH as catalyst, resulting in a very low formation of amide after overnight reaction (0.03 mmol benzylamine/g CNF, entry 7). It clearly demonstrated that AcOH is not the active catalyst. The use of other commercially available catalysts, such as FeCl_2_, Fe_3_Br, (CH_3_C_6_H_4_SO_3_)_3_Fe·6H_2_O, and Fe_2_O_3_, did not improve the incorporation (0.11 to 0.36 mmol benzylamine/g CNF, entries 8–11). When the reaction was conducted using a solvent mixture 1:1 (EtOH:H_2_O) or acetonitrile (CH_3_CN) as the solvent, a reduction in the benzylamine incorporation was evident (entries 12 and 13). Lastly, different reaction time-points were evaluated (from 2 to 8h) when FeCl_3_ was used as the catalyst, observing a linear progression of the reaction overtime (entries 14–17).

**Table 1:**
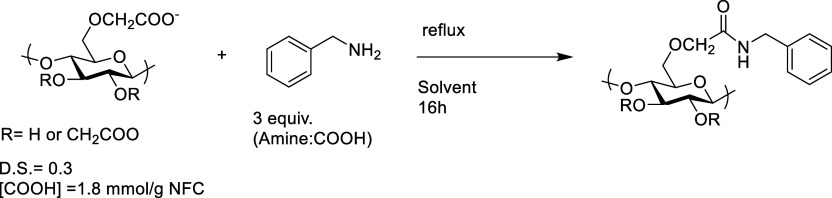
Initial Catalyst Screen in the Amidation Reaction Between CNF and Benzylamine

entry	catalyst (mol %)	additive (equiv)	solvent	temperature	mmol benzylamine/g CNF^e^
1	none	-	PhMe	110 °C	under limit of detection
2	FeCl_3_ (20)	-	PhMe	110 °C	0.38 ± 0.002
3	FeCl_3_ (10)	-	PhMe	110 °C	0.35 ± 0.004
4	FeCl_3_ (10)	AcOH (0.5)	PhMe	110 °C	0.47 ± 0.006
5	FeCl_3_ (5)	AcOH (0.5)	PhMe	110 °C	0.21 ± 0.01
6	FeCl_3_ (10)	AcOH (0.5)	PhMe	50 °C	0.26 ± 0.05
7	none	AcOH	PhMe	110 °C	0.03 ± 0.01
8	FeCl_2_ (10)	AcOH	PhMe	110 °C	0.18 ± 0.04
9	FeBr_3_	AcOH	PhMe	110 °C	0.36 ± 0.03
10	(CH_3_C_6_H_4_SO_3_)_3_Fe·6	AcOH	PhMe	110 °C	0.22 ± 0.06
11	Fe_2_O_3_	-	PhMe	110 °C	0.11 ± 0.01
12	FeCl_3_ (10)	AcOH	EtOH/H_2_O	90 °C	0.17 ± 0.03
13	FeCl_3_ (10)	AcOH	CH_3_CN	85 °C	0.21 ± 0.005
14^a^	FeCl_3_ (10)	AcOH	PhMe	110 °C	0.07 ± 0.01
15^b^	FeCl_3_ (10)	AcOH	PhMe	110 °C	0.11 ± 0.02
16^c^	FeCl_3_ (10)	AcOH	PhMe	110 °C	0.16 ± 0.01
17^d^	FeCl_3_ (10)	AcOH	PhMe	110 °C	0.26 ± 0.03

a After 2 h of reaction.

b After 4 h of reaction.

c After 6 h of reaction.

d After 8 h of reaction.

e By elemental analysis of the total nitrogen content and average of at least three reactions.

**Figure 1:**
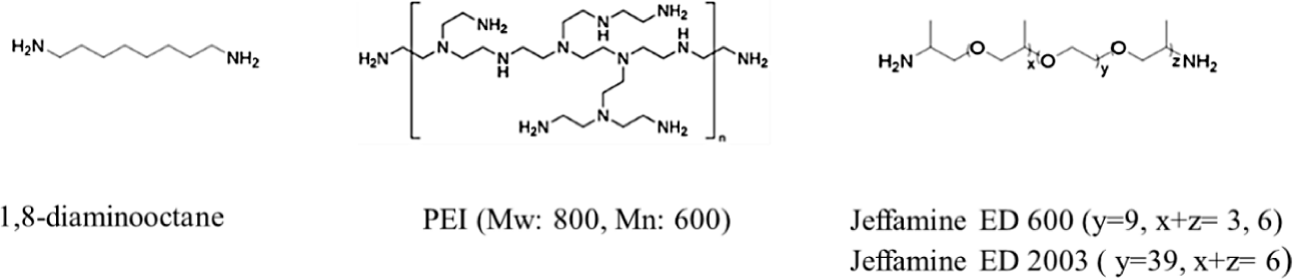
Chemical structure of free-amine-containing molecules used for the substrate scope of the catalytic reactions.

Once the optimal reaction conditions were established (entry 4, Table 1), we proceeded to demonstrate the utility of this catalytic reaction by incorporating synthetic polymers containing aliphatic amines onto CNFs, as depicted in Figure 1. The results of these incorporations are summarized in Table 2. Our experiments included the use of a relatively short aliphatic diamine, 1,8-diaminooctane (entry 1), as well as three distinct polymers featuring free amine groups, namely, PEI, Jeffamine ED 600, and Jeffamine ED 2003 (entries 2–4). The obtained results confirm the versatility of the reaction, enabling the successful grafting of polymers of varying sizes to the CNFs. This capability opens the door to the creation of nanocomposites that hold significant scientific and practical interest.

**Table 2:**
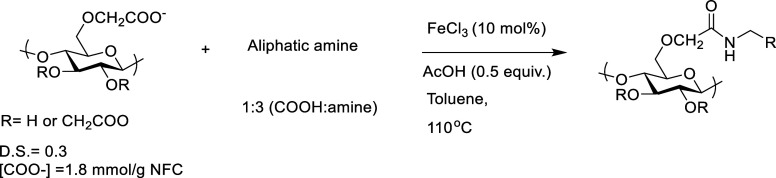
Covalent Incorporation of Valuable Polymers into CNF Using Optimized Catalytic Conditions

entry	amine	mmol copolymer/g CNF^a^
1	1,8-diaminooctane	0.50 ± 0.03
2	PEI (Mw ∼800 g/mol)	0.42 ± 0.02 (**CNF-PEI 800**)
3	Jeffamine ED 600	0.54 ± 0.05 (**CNF-Jeff 600**)
4	Jeffamine ED 2003	0.31 ± 0.01 (**CNF-Jeff 2003**)
5^b^	PEI (Mw ∼800 g/mol)	0.29 ± 0.01
6^c^	PEI (Mw ∼800 g/mol)	0.25 ± 0.06

a By elemental analysis of the total nitrogen content and average of at least three reactions.

b 2-MeTHF as the reaction solvent.

c CPME as the reaction solvent.

Additionally, the production of the CNF-PEI 800 material can be achieved with a more environmentally sustainable approach by substituting toluene with greener solvents. In our investigations, we explored the modification of CNF through the incorporation of PEI using two solvents derived from biomass, specifically 2-MeTHF^52^ and CPME,^53^ as indicated in entries 5 and 6. The catalytic reaction was successful in both cases, although with a decrease in PEI incorporation.

**Figure 2:**
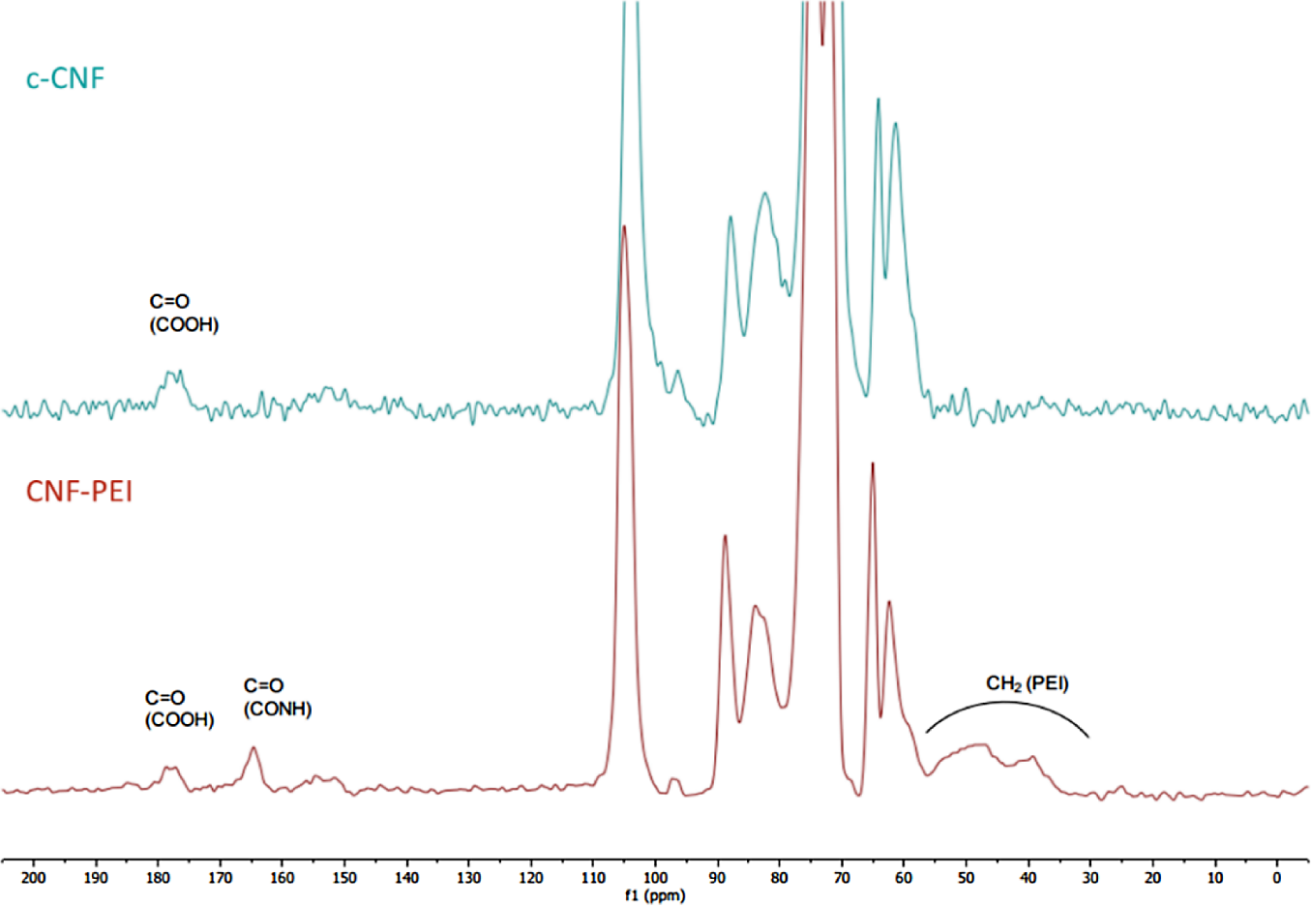
^13^C CP/MAS solid-state nuclear magnetic resonance (CP/MAS ^13^C NMR) spectra of CNF-PEI 800 and c-CNF.

The newly synthesized CNF-PEI 800 nanocomposite material (entry 2, Table 2) was further characterized by CP/MAS ^13^C NMR spectroscopy. The ^13^C NMR spectrum of CNF-PEI 800 is displayed in Figure 2, together with the spectrum of c-CNF as a reference. The signals between 35 and 60 ppm in the CNF-PEI 800 spectrum could be attributed to the methylene groups in PEI. Close inspection of the 160–180 ppm region showed an additional signal (at around 165 ppm) in the carbonyl region due to the formation of the amide bond during the catalytic amidation, and therefore confirms that PEI is covalently bonded to CNF. The largest signals, from 58 to 110 ppm, belong to the cellulose chain carbons (C1 to C6).

In order to shed light about the prevalence of observed catalysis of FeCl_3_ species, theoretical calculations at the ωB97x-D/6-31G(d,p) level of theory were carried out (see Supporting Information for computational details). The preferential coordination of the catalyst was first explored. In all cases, computational outcomes showed that coordination to the carbonyl group was the most stable one. In addition, the carboxymethyl moiety (*gt* anomer) avoided the bidentate coordination of Fe between the C=O group and the endocyclic oxygen of cellulose. However, the role of this oxygen atom proved to be pivotal in assisting the subsequent amine attack through a hydrogen bonding interaction (*R*_O···H2N_ = 2.10 Å, confirmed by NCI plot analysis). In this plot, green surfaces indicate weak intermolecular contacts as expected for hydrogen bonding and related interactions. The energy profile considered that the carbonyl group is preactivated similar to the experimental conditions. In that case, the FeCl_3_ species is already coordinated to the carbonyl oxygen. Using this preactivation as jumping-off point, the optimized transition state (ΔΔ*E* = 12.0 kcal mol^–1^) suggests that is accessible by the temperature in which the reaction was carried out.

**Figure 3:**
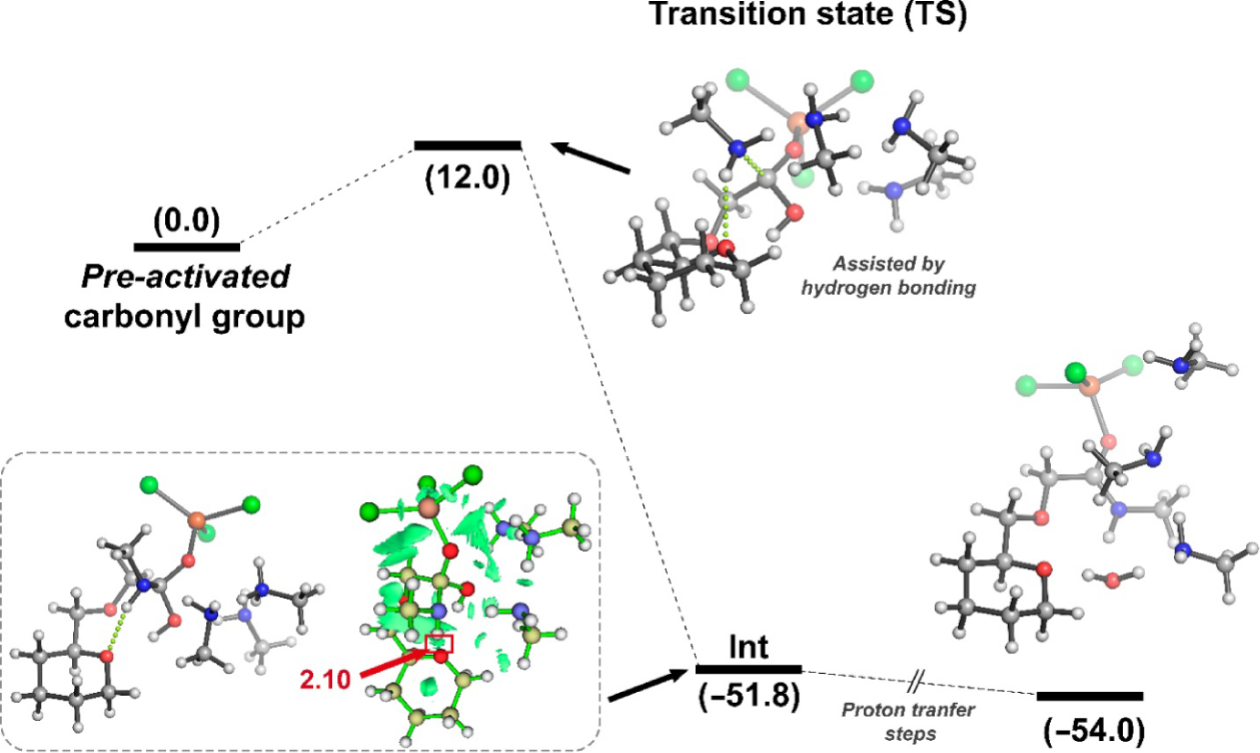
Calculated energy profile of acyl nucleophilic substitution catalyzed by FeCl_3_ at the ωB97x-D/6-31G(d,p) level of theory. The proton transfer steps are not shown. All energies (kcal mol^–1^) are relative to those of reactants. Distances are given in Å.

As expected for a two-step process, the tetrahedral intermediate stabilized by FeCl_3_ coordination was computationally found (ΔΔ*E* = −51.8 kcal mol^–1^). This intermediate evolves toward the final product, realizing a H_2_O molecule. This requires at least three barrierless proton transfer steps (not specified herein). The competition with polar protic solvents makes these steps essentially more difficult due to additional hydrogen bonding with the solvent, in agreement with the catalytic outcome obtained by the EtOH:H_2_O mixture. After that, the DFT results show that the formation of the amide bond is thermodynamically favored (Figure 3, ΔΔ*E* = −54.0 kcal mol^–1^), resulting in the corresponding functionalized cellulose.

As previously noted, the novel materials incorporating PEI are designed for application as coatings to mitigate PJI due to the inherent antimicrobial properties of PEI. Consequently, it is mandatory to assess the adhesive characteristics of these materials for the prosthesis surface. In this study, CNF-PEI 800 was selected as the material of choice for conducting adhesion experiments due to its higher PEI content.

### 3.2 Adhesiveness of CNF-PEI 800 Nanocomposite Material

**Figure 4:**
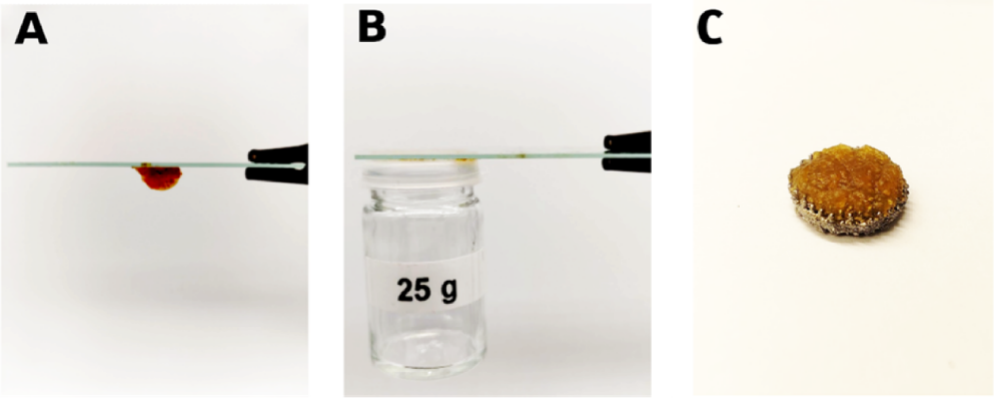
Camera images of the nanocomposite adhesive CNF-PEI 800 hydrogel (1.5 wt % in water, entry 3, Table 2) showed (A) stickiness of the material. (B) Adhesion between dissimilar materials (glass-plastic). (C) Adhesion to trabecular titanium (TrabecuLink) after 3 days of incubation in PBS at 37 °C.

The interest in the development of formaldehyde-free biobased adhesives has increased in recent years due to its toxicity for humans and the environment.^54,55^ In this context, nanocellulose is a material with unique rheological properties, and CNFs have shown adhesion properties previously.^56^ CNF-PEI 800 nanocomposite hydrogel developed in this work presents an increase in the adhesiveness when compared with c-CNF. These adhesive properties increase the potential of CNF-PEI 800 to be used in the field of biomedicine among others.^57^ In the CNF-PEI 800 material, a high amount of positive charges and free amines that can form hydrogen bonds have been introduced.^58,59^ Adhesion between the interfaces of similar and dissimilar materials is highly desirable in various fields. The adhesive properties of the nanocomposite CNF-PEI hydrogel can be observed by the naked eye and were proven by holding several materials together (Figure 4 and Figure S1,). Moreover, CNF-PEI 800 gel was applied to a porous titanium scaffold (TrabecuLink), exhibiting excellent adherence to the scaffold after a 3 days incubation in PBS at 37 °C (Figure 4C). TrabecuLink or trabecular titanium is used clinically in hip and knee reconstruction cases where there is significant bone loss due to prior surgeries.^60,61^

**Figure 5:**
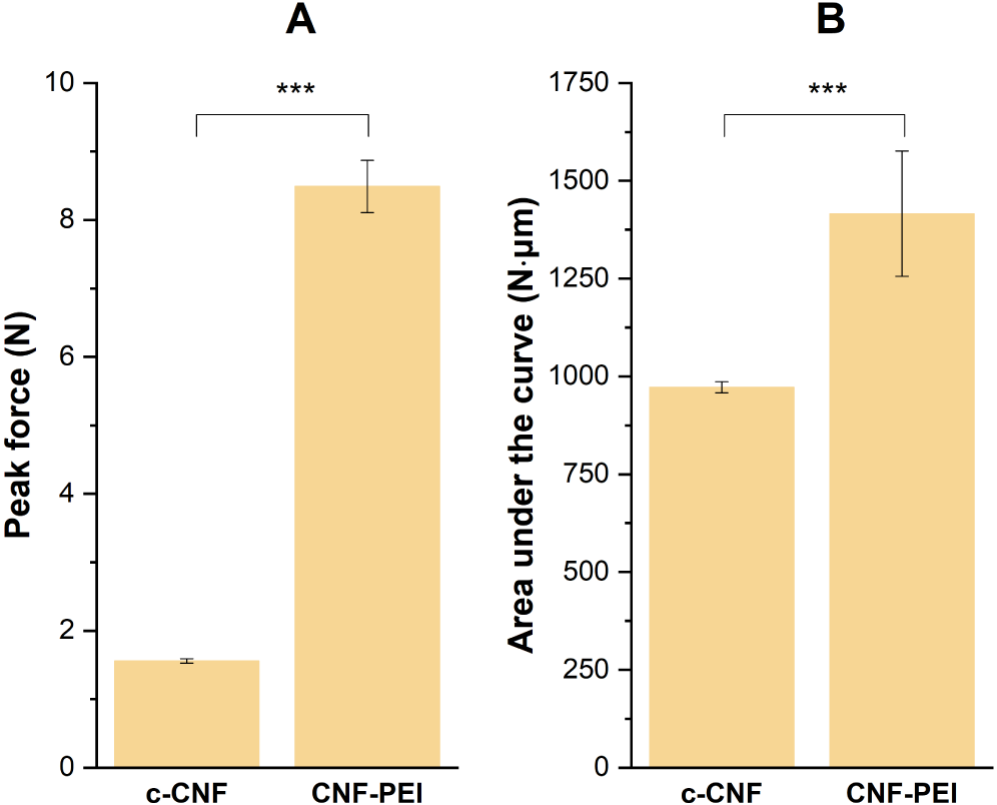
Bar plot of (A) peak force and (B) area under curve from adhesiveness measurements. Error bars correspond to ±1 st. dev. ***Significance level (*p* < 0.0005).

This property of the CNF-PEI 800 hydrogel was further quantified by measuring the adhesiveness (the maximum force needed in the separation process) and work of adhesion (the energy required during the separation process). During adhesiveness measurements, an approximately 5 times higher peak force was observed for the CNF-PEI 800 sample compared to the c-CNF (Figure 5, A). The apparent work of adhesion (N·μm) was evaluated using the area under the force vs gap curves. The difference in work was smaller as compared to the difference in peak force (Figure 5, B). This could be related to the way in which the samples deformed and detached from the surfaces. CPN-PEI 800 showed an adhesive fracture, with the sample abruptly detaching from the upper or lower geometry during measurement. On the other hand, c-CNF showed a cohesive fracture with the sample deforming/flowing to form a liquid bridge between the plates before eventually breaking, leaving sample on both surfaces. (These phenomena are demonstrated in the videos provided in the Supporting Information.) The difference in fracture suggests stronger cohesive forces for CNF-PEI 800 compared to c-CNF.

**Figure 6:**
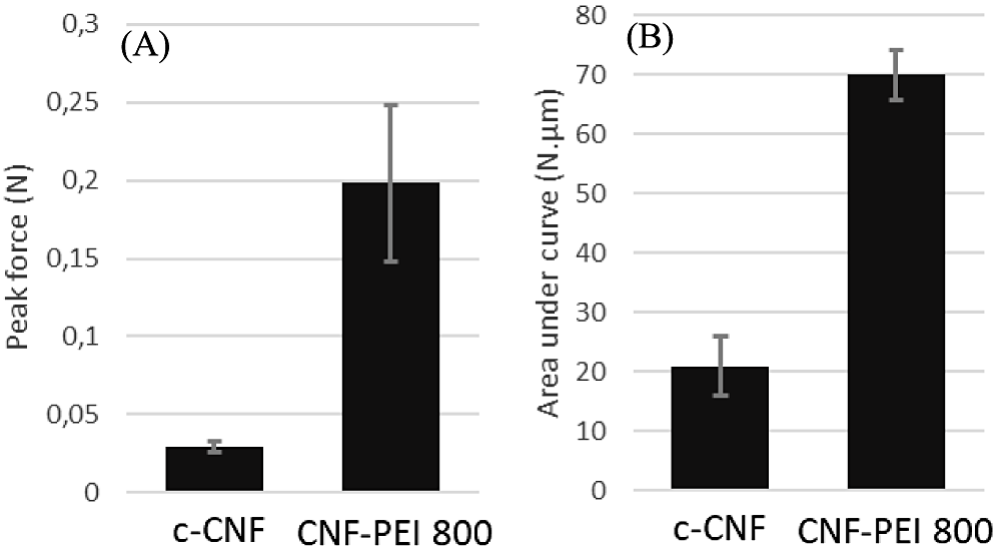
Bar plot of (A) peak force and (B) area under curve from adhesiveness measurements to titanium scaffolds.

As mentioned earlier, the CNF-PEI 800 might have the potential for usage in biomedical applications. To further evaluate this and to confirm the increased adhesiveness also for relevant materials, the adhesiveness to a titanium scaffold was investigated. The results showed a similar trend as for the steel/aluminum geometry, i.e., a higher adhesiveness (approximately 7 times higher) and higher work of adhesion was observed for CNF-PEI-800 compared to c-CNF (Figure 6). The large difference in adhesiveness and work of adhesion compared to the initial measurements using the 40 mm aluminum plate is due to the smaller diameter of the titanium scaffolds (12.5 mm).

**Figure 7:**
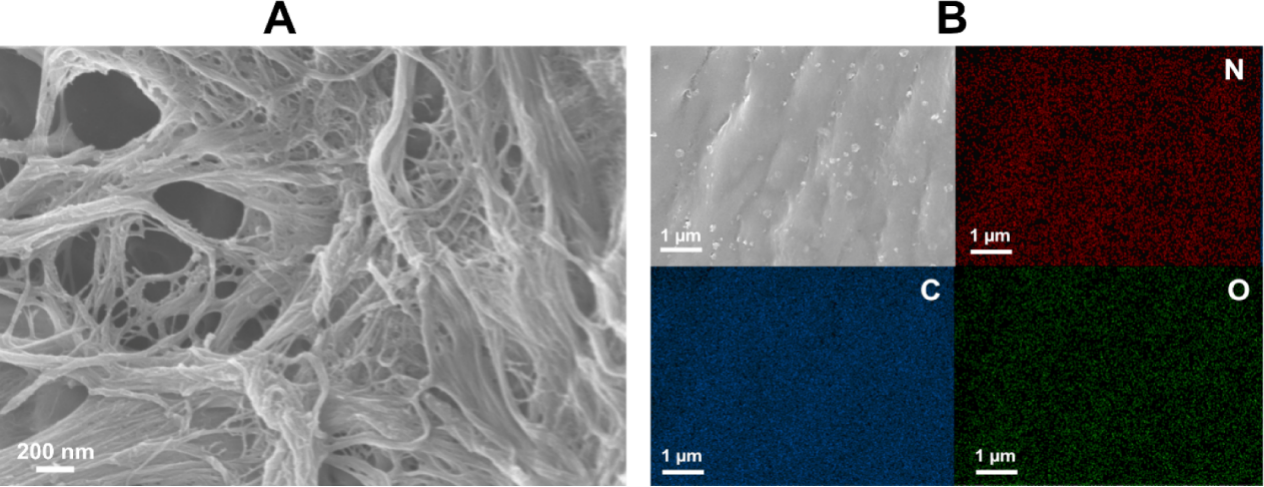
(A) Representative scanning electron microscopy image of a CNF-PEI 800. Scale bars represent 200 nm. (B) Representative images of the scanning of carbon, oxygen, and nitrogen by SEM-EDS of CNF-PEI 800 and the corresponding SEM image (scale bars represent 1 μm).

Additionally, fiber morphology of CNF-PEI 800 was evaluated using SEM, with the images showing the nanofibers structure still remaining after the polymer incorporation (Figure 7A) comparable to the initial c-CNF.^62^ The analysis of the material by SEM-EDS revealed a homogeneous distribution of nitrogen upon the material surface, thus indicating that the covalently bound PEI was uniformly distributed along the fibers (Figure 7B). Besides, Fe was not detectable by EDS after the purification process, verifying that the new properties of the material are nonrelated in the presence of the metal. On the other hand, homogeneous distribution of the metal on the CNF was observed when reaction conditions (110 °C, toluene, overnight) were used and before the purification process (Figure S2,).

Moreover, in CNF-PEI 800, fiber agglomeration appears more pronounced than in c-CNF, likely attributed to two primary factors: covalent cross-linking between fibers and the employment of organic solvents during the catalytic reaction (Figures S3 and S4,).

### 3.3 Osteoblast Cell Cultures

Cell colonization, proliferation, and differentiation are crucial when developing a successful surface modification of an orthopedic implant. This will determine whether the implant successfully integrates with the host bone, and achieving a robust osseointegration is crucial for the implant’s long-term success. Preliminary studies were conducted to assess the biocompatibility of the novel CNF-PEI 800 hydrogel material with hOBs.

**Figure 8:**
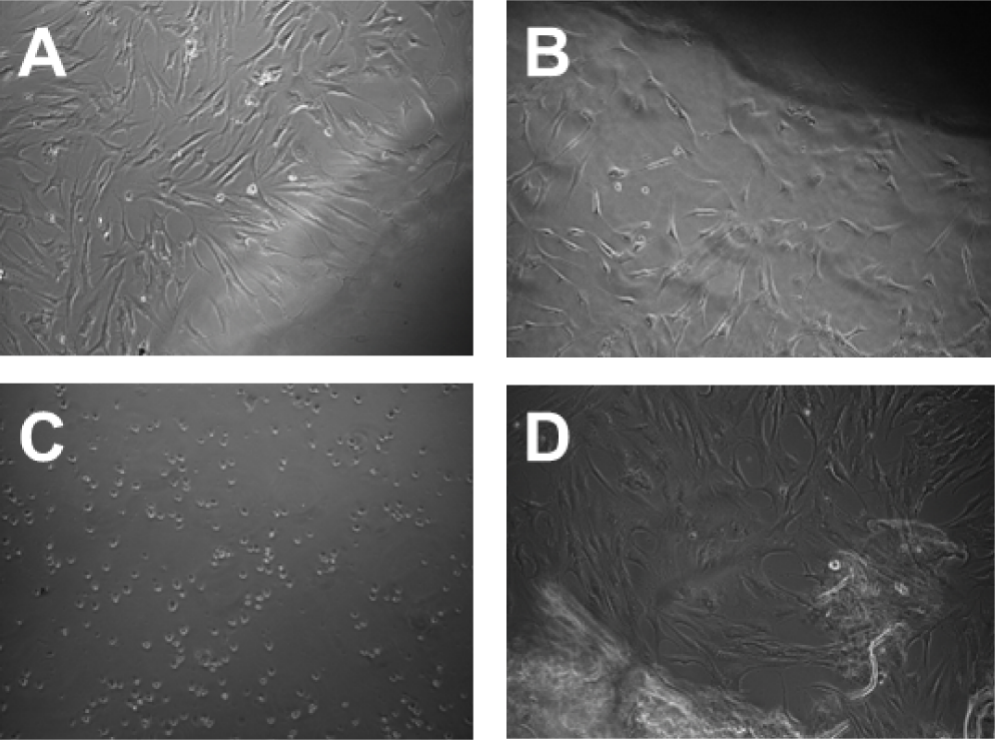
Evaluation of the biocompatibility of the CNF-PEI 800 nanocomposite with hOBs. Optical microscope images of hOBs after incubation: (A) 3 days with CNF-PEI 800, (B) 7 days with CNF-PEI 800, (C) 24 h with PEI, and (D) 7 days with c-CNF.

The biocompatibility of CNF-PEI 800 was evident in our study, with hOBs displaying satisfactory survival after 3 days and 1 week (Figure 8A,B). Significantly, the observed level of compatibility closely parallels that observed with c-CNF (Figure 8D). Besides, the cytotoxicity typically associated with PEI (Figure 8C) was significantly mitigated in the new nanocomposite material.

**Figure 9:**
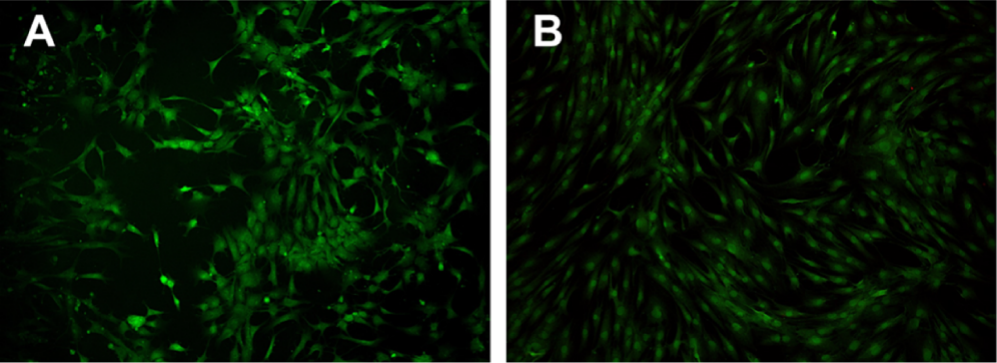
Morphology of hOBs on the (A) CNF-PEI 800 hydrogel and (B) c-CNF hydrogel visualized by staining the cytoplasm with carboxyfluorescein diacetate.

**Figure 10:**
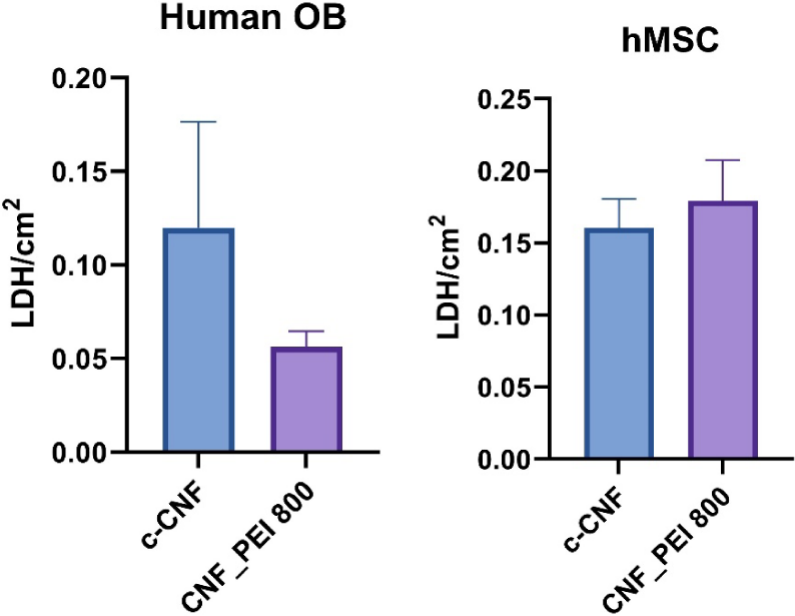
Human OB and hMSC proliferation over a 1 week culture period analyzed by LDH.

Furthermore, both microscopic images after staining the cells (Figure 9A) and LDH values (Figure 10) provided compelling evidence of attachment, growth, and proliferation of hOBs on the CNF-PEI 800 composite hydrogel. However, a decrease in LHD is noted compared to the c-CNF control (Figure 10). Additionally, human mesenchymal stem cells (hMSCs) were cultured on CNF and CNF-PEI 800 materials, showing very similar cell viability after 1 week (Figure 10). This similarity in viability indicates that the introduction of PEI 800 into CNF matrix does not adversely affect cell survival. The materiaís low cytotoxicity and its ability to support cell adhesion, growth, and proliferation make CNF-PEI 800 an interesting material for applications in medical devices, particularly those involved in bone regeneration and repair.

## 4 Conclusion

The present study has demonstrated the application of an efficient and cost-effective catalytic method for the incorporation of polymers into cellulose nanofibers (c-CNF) through direct amidation. The use of a commercially available and inexpensive FeCl_3_ as a catalyst presents notable advantages, including high reaction efficiency and a significant reduction in waste generation. Considering the abundance of amine-containing polymers, it is conceivable that this methodology holds promise for a multitude of applications, extending its relevance beyond the domain of polymer chemistry. Moreover, given the abundant presence of positive charges within this nanocomposite, it holds great potential for loading with drugs or growth factors of interest for specific purposes.

Furthermore, this work demonstrated the generality and versatility of the catalytic approach by developing novel polymer nanocomposites with exciting properties, notably increased adhesiveness and enhanced thermal stability. The catalytic direct amidation has facilitated the synthesis of a CNF-PEI 800 nanocomposite, exhibiting outstanding properties, including improved adhesion and low cytotoxicity against primary osteoblasts. This positions CNF-PEI 800 as a highly promising candidate for coating medical implants. In the future, additional research will be necessary to evaluate both the antibacterial efficacy and immune response associated with the material’s potential as an implant coating.

## Supporting Information Available

Additional camera images of the adhesive nanocomposite CNF-PEI 800 hydrogel; SEM-EDS of Fe distribution on the CNF surface; scheme of covalent and noncovalent interactions in CNF-PEI 800; SEM images of the fibers in c-CNF and CNF-PEI 800; Computational details (PDF)
Video cohesive fracture of hydrogel c-CNF (MP4)
Video adhesive fracture of hydrogel CNF-PEI 800l (MP4)
