# Supplementary material for: A Simple and Cost-Effective FeCl3-Catalyzed Functionalization of Cellulose Nanofibrils: Toward Adhesive Nanocomposite Materials for Medical Implants
Source: ACS Appl Mater Interfaces. 2024 May 31;16(23):30385–95. doi: 10.1021/acsami.4c04351 (PMC11181277; doi:10.1021/acsami.4c04351)
Supplement: Supplementary file 1 — am4c04351_si_001.pdf [file am4c04351_si_001.pdf]

Supporting Information

A Simple and Cost-effective $FeCl_3$-catalyzed Functionalization of Cellulose Nanofibrils: Towards Adhesive Nanocomposite Materials for Medical Implants

*Evgenii Tikhomirov‡,[a] Antonio Franconetti‡,[b] Mathias Johansson,[c] Corine Sandström,[c] Elin Carlsson,[d] Brittmarie Andersson,[d] Nils P Hailer,[d] Natalia Ferraz,[a] Carlos Palo-Nieto[a,d]**

a. a. Nanotechnology and Functional Materials, Department of Materials Science and Engineering, Uppsala University, 751 03, Uppsala, Sweden.
b. Departamento de Química Orgánica, Facultad de Química, Universidad de Sevilla, 41012 Sevilla, Spain.
c. Department of Molecular Sciences, Swedish University of Agricultural Sciences, 756 51, Uppsala, Sweden.
d. Ortholab, Department of Surgical Sciences—Orthopaedics, Uppsala University, 751 85, Uppsala, Sweden.
*Corresponding author: carlos.nieto@uu.se

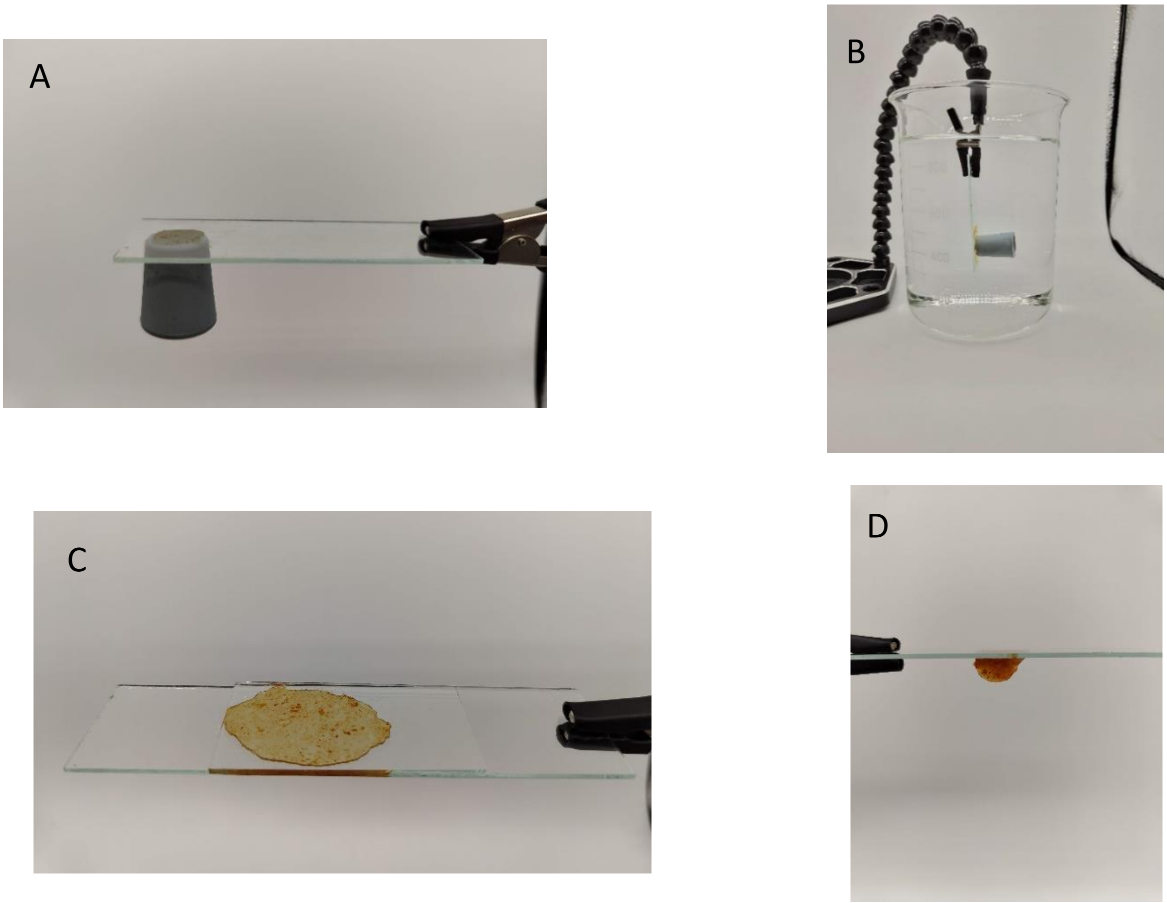

Figure S1. Camera images of the adhesive nanocomposite CNF-PEI 800 hydrogel (1.5 wt% in water, entry 3, table 2) showed that A) promote adhesion between rubber and glass. B) promote adhesion between rubber and glass in water. C) promote adhesion between glass-glass. D) possess adhesive properties.

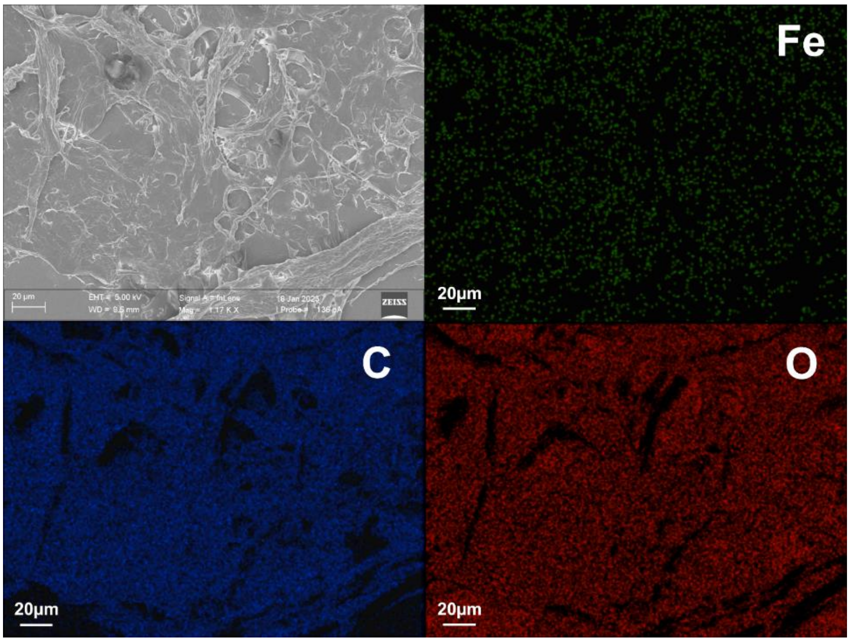

Figure S2. Representative images of the scanning of carbon, oxygen and iron by SEM-EDS of CNF-Fe and the corresponding SEM image (scale bars represent 20 µm).

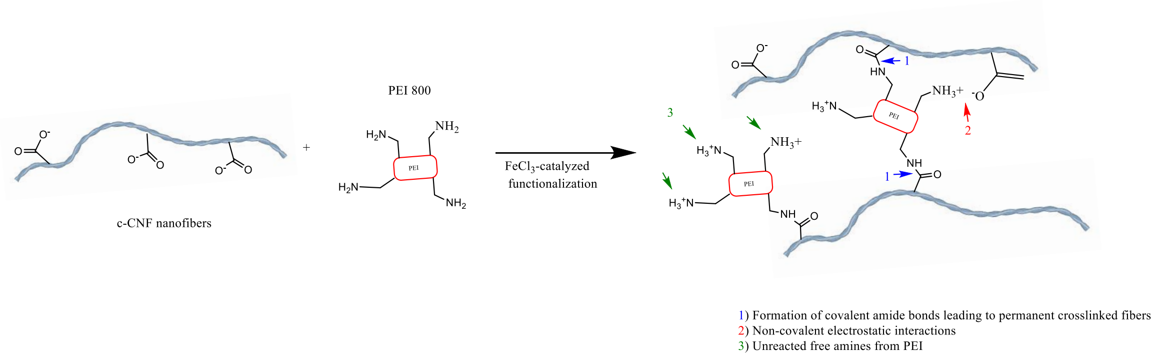

Figure S3. Potential crosslinking interactions between carboxylic acid c-CNF fibers and amines from PEI polymer.

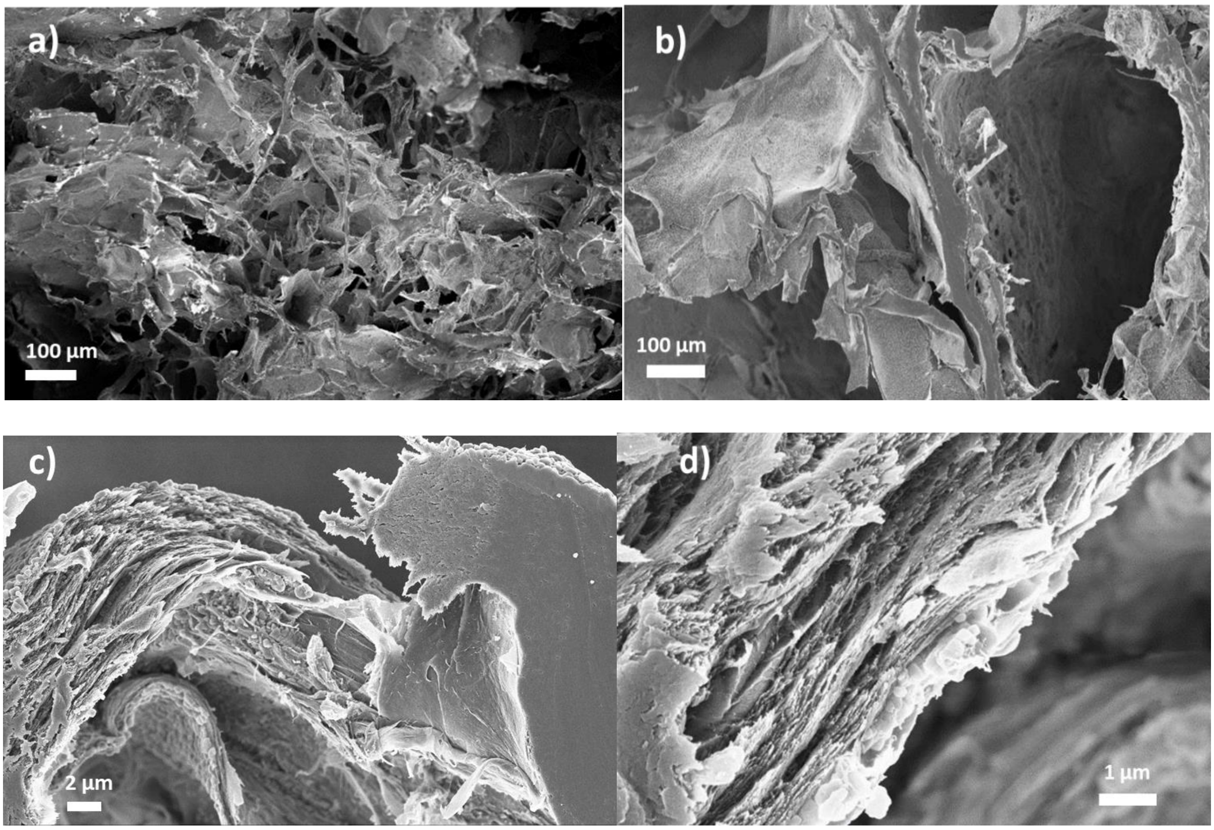

Figure S4. Representative scanning electron microscopy images of a) c-CNF fibers, b) CNF-PEI 800 fibers and c), d) cross-section CNF-PEI 800.

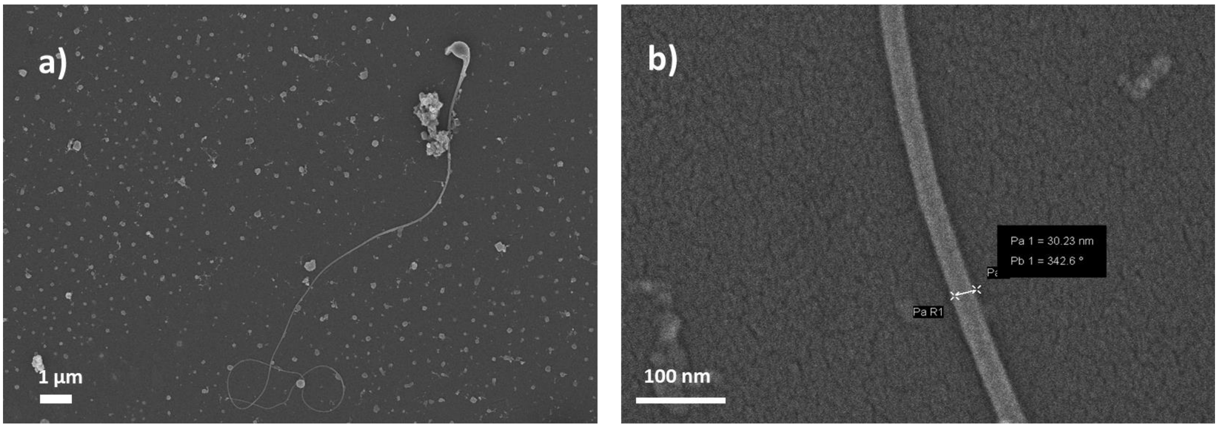

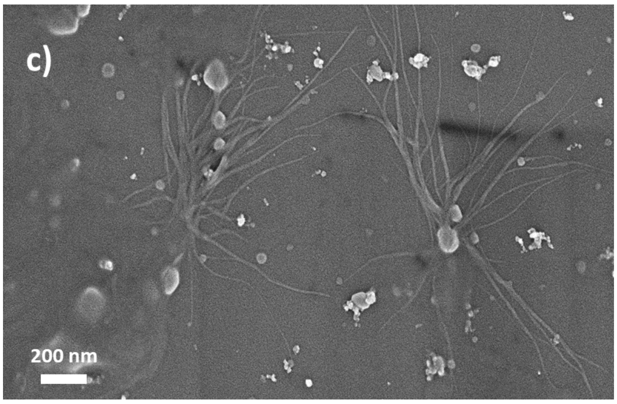

Figure S5. Representative scanning electron microscopy images of a), b) c-CNF single fiber (width=30-35 nm and length= several microns, c) CNF-PEI 800 fibers exhibit similar width and length, yet they are agglomerated.

2.5 Computational details

Theoretical calculations were performed the Gaussian 16 program. [1] Geometry optimizations were carried out using ωB97x-D[2] and 6-31G(d,p) basis set. In order to characterize the nature (minimum or transition state) of optimized structure, analyses of frequencies were also conducted. Minima are described by absence of imaginary frequencies whereas one imaginary frequency was obtained for transition states. Implicit solvent effects (toluene) were considered applying the IEF-PCM polarizable continuum model[3] by means of single-point calculations. In addition to consider solvent effects, we have used four implicit amine molecules in the theoretical models. The utilization of these molecules is in agreement with the experimental conditions and they are necessary to account the proton transfer steps along the reaction pathway. In all cases, methylamine was employed as computational model. In addition, only the substituent of C-5 of the glucoside moiety was considered. NCIplot isosurfaces[4] were computed using cubefiles (medium quality grid) generated from Multiwfn v. 3.8 program.[5] The color scheme for studying weak non-covalent interactions depends on the value of the reduced density gradient. In general, the colour codes are red (repulsive, ρ^{+}_{cut}), and blue (attractive, ρ^{-}_{cut}) whereas yellow-green surfaces indicate weak repulsive and weak attractive, respectively.

References

[1] Gaussian 16, Revision C.01, Frisch, M. J.; Trucks, G. W.; Schlegel, H. B.; Scuseria, G. E.; Robb, M. A.; Cheeseman, J. R.; Scalmani, G.; Barone, V.; Petersson, G. A.; Nakatsuji, H.; Li, X.; Caricato, M.; Marenich, A. V.; Bloino, J.; Janesko, B. G.; Gomperts, R.; Mennucci, B.; Hratchian, H. P.; Ortiz, J. V.; Izmaylov, A. F.; Sonnenberg, J. L.; Williams-Young, D.; Ding, F.; Lipparini, F.; Egidi, F.; Goings, J.; Peng, B.; Petrone, A.; Henderson, T.; Ranasinghe, D.; Zakrzewski, V. G.; Gao, J.; Rega, N.; Zheng, G.; Liang, W.; Hada, M.; Ehara, M.; Toyota, K.; Fukuda, R.; Hasegawa, J.; Ishida, M.; Nakajima, T.; Honda, Y.; Kitao, O.; Nakai, H.; Vreven, T.; Throssell, K.; Montgomery, J. A., Jr.; Peralta, J. E.; Ogliaro, F.; Bearpark, M. J.; Heyd, J. J.; Brothers, E. N.; Kudin, K. N.; Staroverov, V. N.; Keith, T. A.; Kobayashi, R.; Normand, J.; Raghavachari, K.; Rendell, A. P.; Burant, J. C.; Iyengar, S. S.; Tomasi, J.; Cossi, M.; Millam, J. M.; Klene, M.; Adamo, C.; Cammi, R.; Ochterski, J. W.; Martin, R. L.; Morokuma, K.; Farkas, O.; Foresman, J. B.; Fox, D. J. Gaussian, Inc., Wallingford CT, 2016.

[2] Chai, J.-D.; Head-Gordon, M. Long-range corrected hybrid density functionals with damped atom–atom dispersion corrections. *Phys. Chem. Chem. Phys.* **2008**, *10*, 6615–6620.

[3] Scalmani, G.; Frisch, M. J. Continuous surface charge polarizable continuum models of solvation. I. General formalism. *J. Chem. Phys.* **2010**, *132*, 114110.

[4] (a) Contreras-García, J.; Johnson, E. R.; Keinan, S.; Chaudret, R.; Piquemal, J. –P.; Beratan, D. N.; Yang, W. NCIPLOT: A Program for Plotting Noncovalent Interaction Regions. *J. Chem. Theory Comput.* **2011**, *7*, 625-632; (b) Johnson, E. R.; Keinan, S.; Mori-Sánchez, P.; Contreras-García, J.; Cohen, A. J.; Yang, W. Revealing Noncovalent Interactions. *J. Am. Chem. Soc.* **2010**, *132*, 6498-6506.

[5] Lu, T.; Chen, F. Multiwfn: A multifunctional wavefunction analyser. *J. Comput. Chem.* **2012**, *33*, 580-592.

[6] De Keer, L. et al.: Computational prediction of the molecular configuration of three-dimensional network polymers *Nat. Mater.* **2021**, *20*, 1422.
